# Bile acids and their respective conjugates elicit different responses in neonatal cardiomyocytes: role of Gi protein, muscarinic receptors and TGR5

**DOI:** 10.1038/s41598-018-25569-4

**Published:** 2018-05-08

**Authors:** Effendi Ibrahim, Ivan Diakonov, Dulasi Arunthavarajah, Teresa Swift, Mary Goodwin, Saraid McIlvride, Vanya Nikolova, Catherine Williamson, Julia Gorelik

**Affiliations:** 1 National Heart and Lung Institute, Imperial College London, London, W12 0NN UK; 2 Kings College London, London, UK; 3 Faculty of Medicine, MARA University of Technology, Sungai Buloh, Malaysia

## Abstract

Bile acids are recognised as bioactive signalling molecules. While they are known to influence arrhythmia susceptibility in cholestasis, there is limited knowledge about the underlying mechanisms. To delineate mechanisms underlying fetal heart rhythm disturbances in cholestatic pregnancy, we used FRET microscopy to monitor cAMP release and contraction measurements in isolated rodent neonatal cardiomyocytes. The unconjugated bile acids CDCA, DCA and UDCA and, to a lesser extent, CA were found to be relatively potent agonists for the GPBAR1 (TGR5) receptor and elicit cAMP release, whereas all glyco- and tauro- conjugated bile acids are weak agonists. The bile acid-induced cAMP production does not lead to an increase in contraction rate, and seems to be mediated by the RI isoform of adenylate cyclase, unlike adrenaline-dependent release which is mediated by the RII isoform. In contrast, bile acids elicited slowing of neonatal cardiomyocyte contraction indicating that other signalling pathways are involved. The conjugated bile acids were found to be partial agonists of the muscarinic M_2,_ but not sphingosin-1-phosphate-2, receptors, and act partially through the G_i_ pathway. Furthermore, the contraction slowing effect of unconjugated bile acids may also relate to cytotoxicity at higher concentrations.

## Introduction

Intrahepatic cholestasis of pregnancy (ICP) is a gestational liver disorder that typically presents in the third trimester of pregnancy. ICP affects 0.5–1% of pregnant women in Europe, and up to 5% in Chile^1,2^. Characterised by maternal pruritus, abnormal liver function and increased maternal serum bile acids, ICP is complicated by increased rates of adverse pregnancy outcomes, including spontaneous preterm labour and stillbirth^3,4^. Furthermore, abnormalities in fetal cardiac rhythm have been reported using echocardiography, including atrial flutter and supraventricular tachycardia, and *in vitro* models have indicated that these may be explained by elevated fetal serum bile acids^5–7^.

Bile acids are cholesterol derivatives, and function as signalling molecules that influence lipid, glucose and energy metabolism as well as immune responses^8,9^. Bile acids can activate nuclear receptors, in particular the farnesoid X receptor (FXR), but also pregnane X receptor (PXR) and vitamin D receptor (VDR), as well as cell surface G-protein coupled receptors, among which the major receptor is GPBAR1 (TGR5)^10^.

In mice, 95% of bile acids are conjugated to taurine rather than glycine, while the glycine:taurine conjugation ratio in humans is 3:1^9^. In cholestatic disorders there is deranged bile acid homeostasis, resulting in bile acid accumulation in the circulation. In ICP, the principal bile acids elevated in the maternal serum are the tauro- and glyco-conjugates of primary bile acids, cholic (CA) and chenodeoxycholic acid (CDCA)^11^, which can be markedly elevated, up to several hundred fold. While serum bile acid concentrations of ≥40 µmol/L are associated with adverse pregnancy outcomes, it has been shown that as maternal serum concentrations of bile acids rise further, and concentrations as high as 500 µmol/L have been reported, the risk of adverse outcomes increases^12,13^. The concentration of bile acids in the fetal serum is typically lower than in maternal serum, but they are both raised in ICP, and in untreated pregnancies the principal fetal bile acids are tauro- and glyco-conjugates of CA and CDCA^14^. Following ursodeoxycholic acid (UDCA) treatment, the proportion of unconjugated bile acids in fetal serum increases, and this is primarily explained by higher concentrations of unconjugated UDCA^11^.

It was proposed that ICP-associated fetal arrhythmia and intrauterine death may result from raised tauro-conjugated bile acids^6^, and previous work has focussed on the arrhythmogenic effects of TCA on cardiomyocytes and myofibroblasts^15,16^. However, TCA is not the only bile acid to be elevated in maternal and fetal serum, and the impact of other bile acids on fetal arrhythmias has not been established.

UDCA is currently used as a treatment of ICP and can improve pruritus and decrease bile acid concentrations in the maternal blood^17–20^. Some studies have suggested that UDCA may improve the rate of adverse pregnancy outcomes^21^, although this has not yet been tested in a prospective, randomised, controlled trial. We have previously reported that UDCA protects against taurocholic acid-(TCA)-induced arrhythmia in a model of the fetal heart focussed on the role of myofibroblasts^16^, but the precise mechanism by which UDCA functions in cardiomyocytes remains poorly understood.

GPBAR1 (TGR5), the Gαs protein-coupled receptor, was the first recognised plasma membrane receptor for bile acids; CHO cells transfected with TGR5 were treated with conjugated and unconjugated bile acids, and the production of cAMP was noted^22,23^. TGR5 is expressed in the hearts of mice and humans, but its role in myocardial cell biology and stress response is unknown. Recently it has been shown that cardiomyocyte specific TGR5 deleted mice have impaired exercise tolerance, catecholamine response under stress, and increased mortality and contractile failure following Transverse Aortic Constriction (TAC)^24^.

Bile acids can also interact with muscarinic cholinergic receptors^25^. Chronotropic effects (changes in contraction rate) TCA on neonatal cardiomyocytes have been shown to be partially eliminated by the pharmacological inhibition of the muscarinic M2 receptor, and TCA’s ability to stimulate Gi like effects on cardiomyocytes has also been previously shown^15^.

S1P receptors have been shown to be expressed in cardiac tissue; while S1P1 and, to a lesser degree, S1P3 are expressed in adult cardiomyocytes, S1P2 is not highly expressed^26,27^. Although mice deficient in S1P2 exhibit no phenotypic effects, except sporadic seizures; it has been demonstrated that conjugated bile acids induce S1P2 in primary rodent hepatocytes, consequently stimulating downstream ERK 1/2 and AKT cascade^28,29^.

Increasing concentrations of relatively hydrophobic BAs have demonstrated cytotoxic behaviour by inducing mitochondrial membrane permeability transition (MMPT) in rat hepatocytes, suggesting that MMPT is a pivotal early stage marker of apoptosis and necrosis^30,31^. UDCA is shown to protect from GDCA-induced MMPT, which can, in part, explain the cytoprotective effect of UDCA in the liver^32^.

In this study, we have comprehensively explored the signalling role of conjugated and unconjugated bile acids via bile acid receptors, and the mechanism by which bile acids may induce chronotropic effects in cardiovascular tissues. It was found that DCA and its conjugates taurodeoxycholic acid (TDCA) and glycodeoxycholic acid (GDCA) elicited slowing of neonatal cardiomyocyte contraction, although only DCA induced a high cAMP release. TDCA and GDCA were found to be partial agonists of the muscarinic M2 receptor that mediate their arrhythmogenic effects partially through the Gi pathway.

## Methods

### Reagents and animals

Bile acids were purchased from Sigma-Aldrich (Haverhill, UK); the specific TGR5 agonist INT-777 was kindly provided by Intercept Pharmaceuticals (New York, USA).

All animals were cared for and housed in accordance with the United Kingdom Home Office Guide on Animals (Scientific Procedures) Act 1986 and local ethical guidelines (project licence PEE7C76CD). Animals were purchased from Harlan Laboratories, UK.

### Neonatal Mouse Cardiomyocytes

Neonatal mouse ventricular cardiomyocytes (NMVMs) were isolated, using the MACS Neonatal Heart Dissociation Kit (Miltenyi Biotec Woking, UK), from the hearts of two-three day old, cEpac1-camps transgenic, Tgr5-KO and wild type C57BL/6 mice. Cells were maintained in plating medium (Medium M199 with Hank’s salts, 5% fetal calf serum (v/v), L-glutamine, Vitamin B12, 200 µg/ml streptomycin, 200 U/ml penicillin). Cells were incubated in 19% oxygen (O_2_), 1% carbon dioxide (CO_2_) and humidified incubator at 37 °C, for three days, at which point confluent networks of cardiomyocytes would have been formed.

### FRET measurement of cAMP production

cAMP production was measured using FRET imaging in transgenic NMVMs expressing cAMP sensor cEpac1-camps at room temperature after washing with FRET buffer (144 mM NaCl, 5.4 mM KCl, 1 mM MgCl_2_, 2 mM CaCl_2_, and 10 mM HEPES, pH = 7.3.) to establish a baseline. NMVMs were incubated with bile acids and then adenylate cyclase activator NKH477 (NKH) to establish maximum cAMP production. The FRET imaging system consisted of a Nikon Eclipse TE200-U microscope (Nikon Corporation), a mercury lamp (HB0103W/2, Osram), filter cube with EX436/20 excitation filter and DM455 dichroic mirror (Chroma), a 60× objective lens, a Dualview beam splitter equipped with 535/40 and 480/30 emission filters, to separate cell fluorescence into YFP and CFP channels (Photometrics). Time-lapse imaging was executed with a Hamamatsu ORCA-ER (Hamamatsu) camera with a frame every 5 seconds. MicroManager 1. 4 software (Open Imaging) was used for FRET data capture and analysis. FRET ratio was calculated by dividing YFP intensity to CFP intensity and kinetic traces were plotted normalised to baseline for each experiment, relative cAMP production was calculated by normalising FRET ratio following bile acid incubation (100 µM) to the maximum cAMP release following NKH incubation.

### Contraction

Beating frequencies in clusters of NMVMs (about 20,000 cells) were manually recorded under a light microscope in accordance with Clark *et al*. 1991^33^. Contraction of 10 cell clusters/area per condition was counted before (vehicle) and after treatment with increasing concentrations of bile acids at 10 µM, 50 µM and 100 µM for 15 minutes and then Hank’s salts (HBSS) for 15 minutes. In some experiments NMVMs were incubated with 1.5 µg/ml pertussis toxin (PTX) for 3 hours, 1 µmol/L methoctramine for 30 minutes or 10 µmol/L JTE 013 (Tocris Bioscience) for 30 minutes before bile acid treatment.

### Cytotoxicity

NMVMs were plated in a 96 well plate at 50,000 cells per well. Cells were treated with BAs at varying concentrations for 24 hours. Treatment with 5 µM Doxorubicin (DOX) was used as positive controls for cell death. NMVMs were then stained with Hoeschst 33342 (0.5 µg/ml, ThermoFisher) and TMRM (20 nM, ThermoFisher). Cells were imaged and analysed with ArrayScanV automatic microscope (Cellomics, ThermoFisher).

### Statistics

FRET data were analysed with one-way ANOVA with Bonferroni post-hoc analysis and, where appropriate, T-test. Data is presented as mean ± SEM. Contraction measurements were analysed with two-way ANOVA with Bonferroni post-hoc analysis. Data is presented as mean ± SEM. Cytotoxicity data were analysed with one-way ANOVA. Data is presented as mean ± SEM.

### Data availability

The datasets generated during and/or analysed during the current study are available from the corresponding author on reasonable request.

## Results

### Un-conjugated bile acids, but not conjugated bile acids, induce high cAMP response in NMVMs

**Figure 1:**
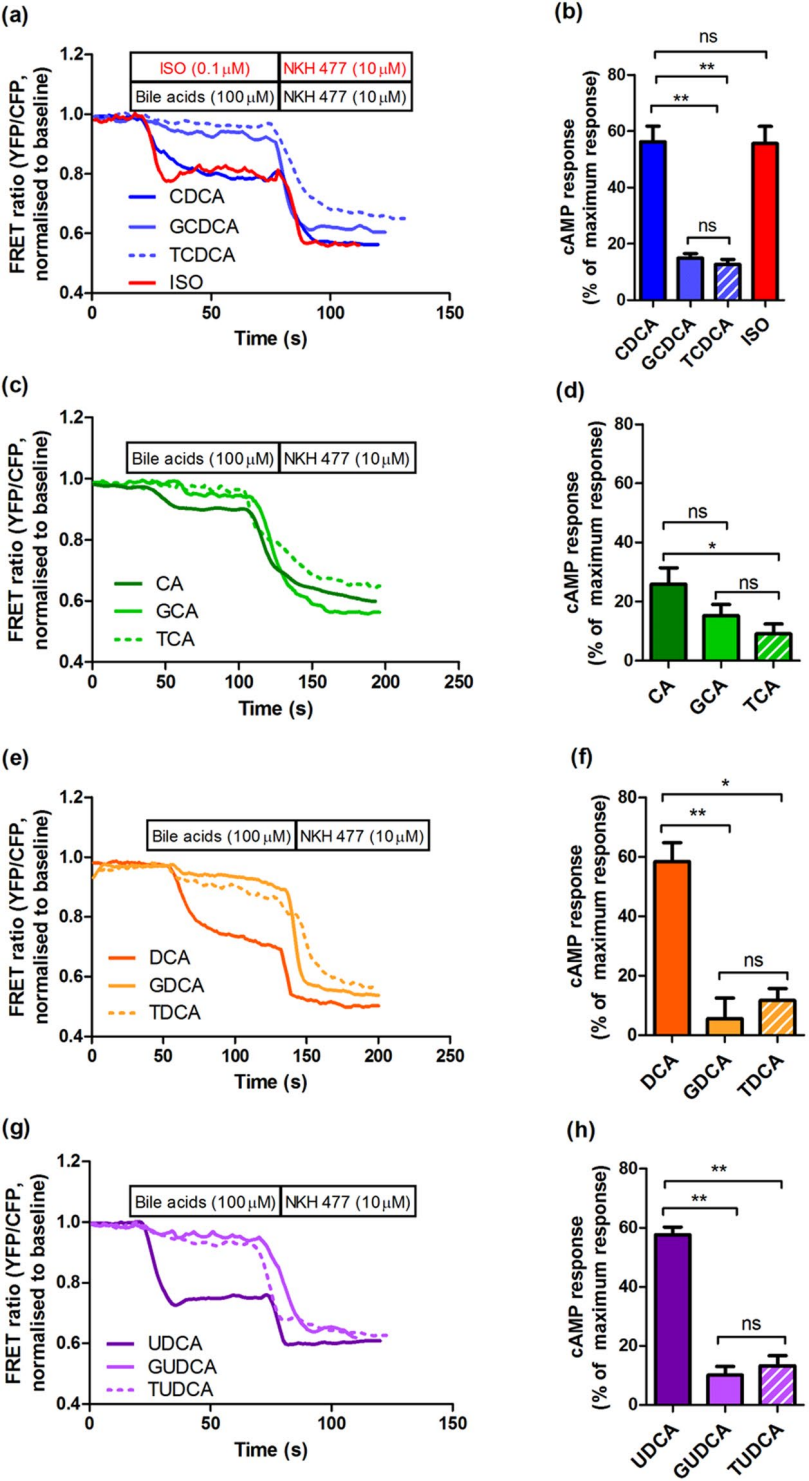
Unconjugated bile acids, but not their respective conjugates, elicit cAMP production in NMVMs. Representative traces of FRET in neonatal mouse cardiomyocytes (NMVMs) of cEpac-camps transgenic mice treated with (**a**) CDCA, (**c**) CA, (**e**) DCA and (**f**) UDCA with their respective tauro- and glyco-conjugates at 100 μM; In (**a**) and (**b**) the cAMP response to isoproterenol (ISO) is also shown; Normalised cAMP responses of NMVMs following stimulation of (**b**) CDCA, (**d**) CA, (**f**) DCA and (**h**) UDCA with their respective tauro- and glyco-conjugates at 100 μM (n = 6, *p < 0.05, **p < 0.01).

**Figure 2:**
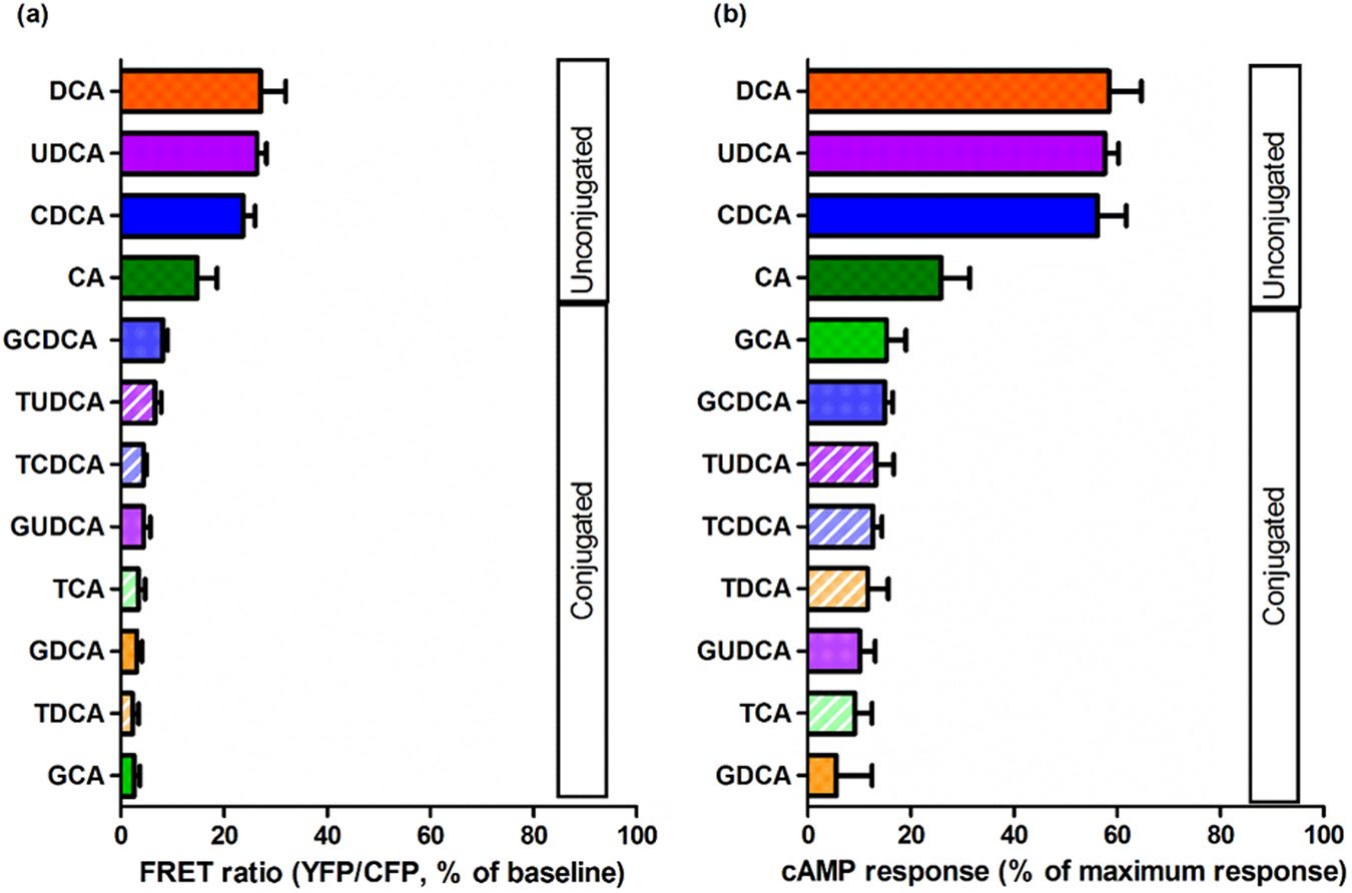
Bile acids and their conjugates ranked by their potency in inducing cAMP responses in NRVMs following stimulation at 100 µM.

The unconjugated bile acids CDCA, DCA and UDCA induce a large cAMP response in NMVMs of 56.19% ± 5.55, 56.79% ± 17.55, and 57.65% ± 2.63, respectively (Fig. 1b,f,h); in contrast, CA induces a lower cAMP response at 17.02% ± 4.89 (Fig. 1d). The responses to CDCA, DCA and UDCA were comparable with the cAMP response following isoproterenol incubation (a gold standard reference compound for GPCR cAMP response, Fig. 1a and b). All tauro- and glyco- conjugated bile acids were much less effective in eliciting cAMP response (range 5 to 15%) than their unconjugated counterparts, except for CA (Fig. 1). Figure 2 summarises the potency of different bile acids at inducing cAMP response in NMVMs following acute incubation with 100 μM bile acids.

### Bile acid receptor GPBAR1 (TGR5) elicits cAMP response in NMVMs

Analysis of mRNA expression with QRT-PCR shows that TGR5 is expressed in both adult and neonatal isolated mouse cardiomyocytes (Figure S1a). Moreover, in neonates the expression is very substantial, comparable with adult spleen, whereas adult cardiomyocytes express much less TGR5. However, the overall expression of TGR5 was low relative to highly expressed genes, e.g. ribosomal protein RPL19, used as housekeeping reference gene. PCR analysis was used also to validate our TGR5KO mouse model and confirmed low TGR5 expression. Similarly, TGR5 expression was detected in human fetal myocytes, and to a lesser extent in human adult cardiac myocytes (Figure S1b).

**Figure 3:**
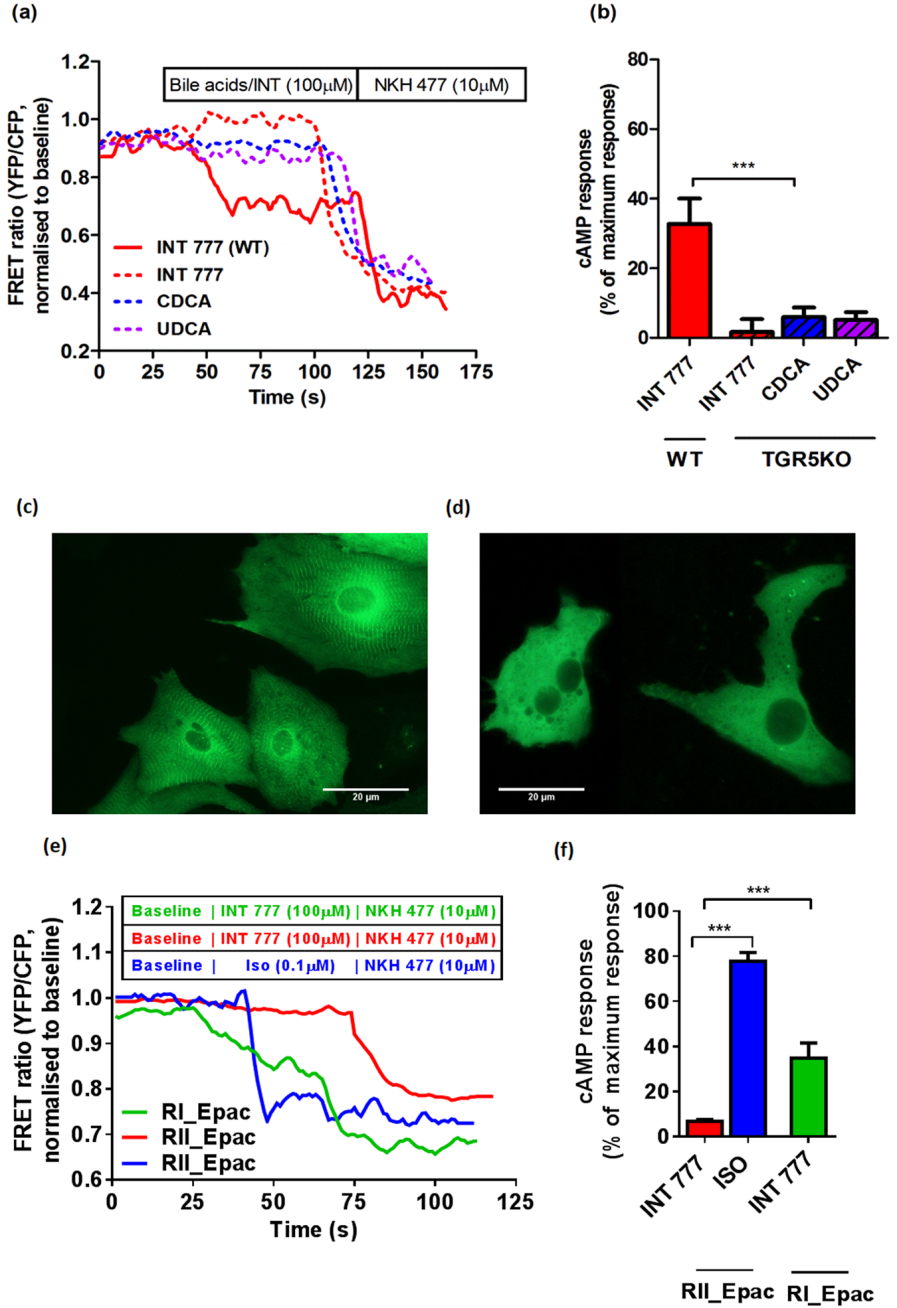
Lack of TGR5 abolishes cAMP response to bile acids in NRVMs. (**a**) Representative FRET traces of TGR5KO neonatal mouse cardiomyocytes treated with CDCA and UDCA at 100 μM. (**b**) Normalised cAMP responses of TGR5KO neonatal mouse cardiomyocytes following stimulation with CDCA and UDCA at 100 μM (n = 6). Adenylate cyclase isoform specificity in cAMP production following TGR5 stimulation Representative images of fluorescent neonatal rat cardiomyocytes transfected with RII-Epac FRET sensor (**c**) and RI-Epac sensor (**d**); (**e**) Representative FRET tracing of neonatal rat cardiomyocytes transfected with Ad-RII-Epac sensor following stimulation with 100 μM INT 777 (red line) and 0.1 μM Isoprenaline (blue line) and transfected with Ad-RI-Epac sensor following stimulation with 100 μM INT 777 (green line); (**f**) % of cAMP response in neonatal rat cardiomyocytes treated as in (**e**) (n ≥ 10, ***p < 0.0001, Unpaired t-test). Data presented as mean ± SEM.

A semisynthetic CA derivative, 6α-ethyl-23(S)-methylcholic acid (S-EMCA, INT-777), has been previously shown to be a potent and specific TGR5 agonist^34^. Indeed, INT-777 generates a cAMP response in NMVMs (Fig. 3a,b). In contrast, no response to INT-777 was seen in NMVMs isolated from TGR5KO mice (Fig. 3a,b). Furthermore, the effect of CDCA and UDCA on cAMP response was completely abolished in TGR5KO NMVMs, indicating that unconjugated bile acids are TGR5 agonists in this ventricular myocyte cell culture (Fig. 3a,b).

**Figure 4:**
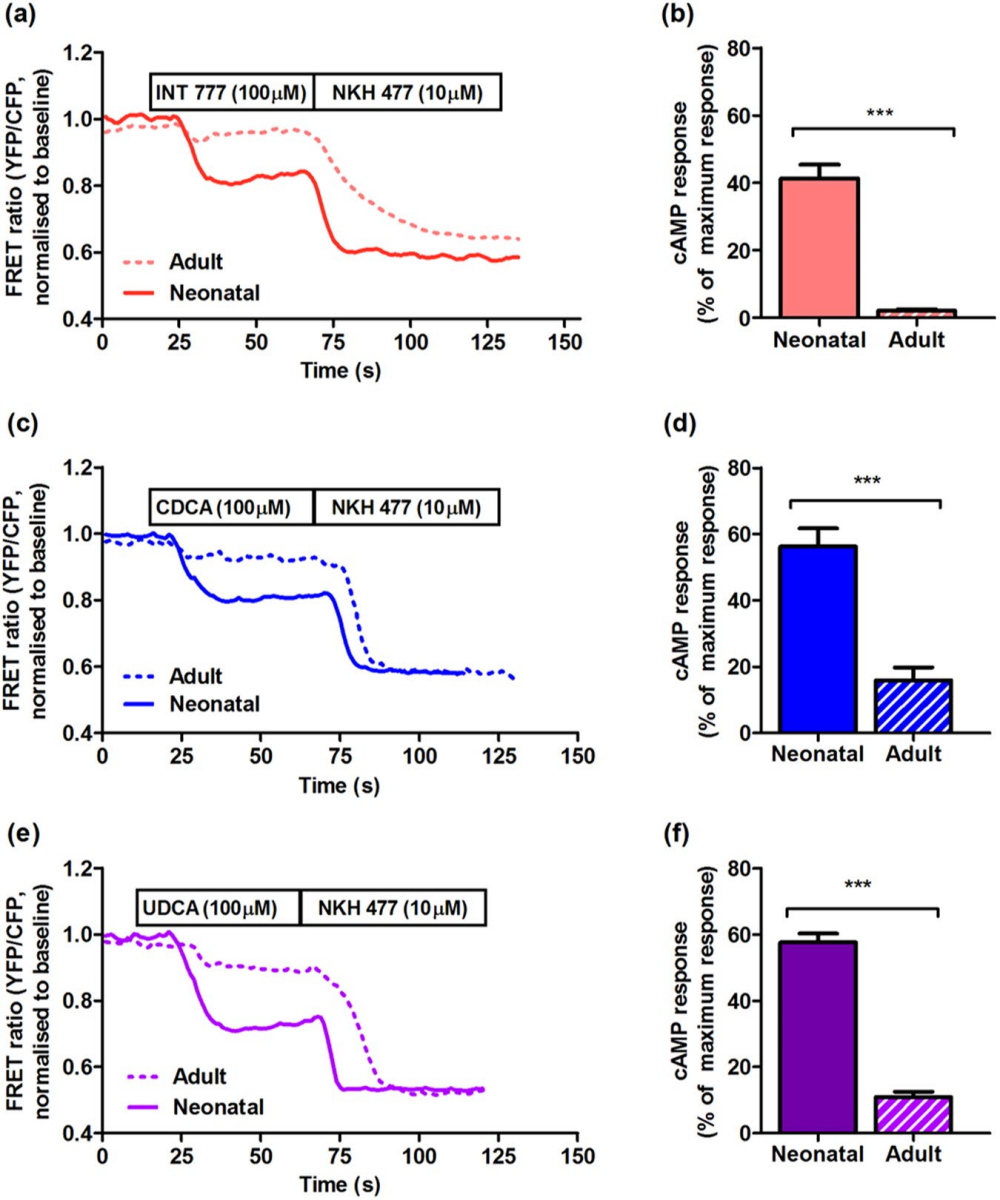
Adult cardiomyocytes show less cAMP response for bile acids compared to neonatal. Representative traces of FRET in neonatal and adult mouse cardiomyocytes of cEpac1-camps transgenic mice treated with (**a**) INT777, (**c**) CDCA, (**e**) UDCA at 100 μM; Normalised cAMP responses of neonatal and adult cardiomyocytes following stimulation of (**b**) INT777, (**d**) CDCA, (**f**) UDCA at 100 μM (n = 6, *p < 0.05, **p < 0.01).

However, the release of cAMP does not translate to an increase of contraction rate (Fig. 4c), which is normally seen following cAMP release induced with isoproterenol^35^. To address the differences in downstream signalling following stimulation with INT-777 and isoproterenol, we used FRET sensors specifically targeted to cellular compartments containing different isoforms of adenylate cyclase (AC)^35^. NMVMs transfected with the sensor localised to the RII isoform of AC show the striated pattern, as expected (Fig. 3c), whereas cells transfected with sensor localised to the RI isoform have a diffuse pattern (Fig. 3d). As anticipated, stimulation with isoprenaline elicits RIIAC activity; in contrast, no FRET signal was detected following stimulation with INT-777. Moreover, the INT-777-dependent cAMP release was detected with RIAC-targeted sensor (Fig. 3e,f).

The response to INT-777 is more pronounced in neonatal cardiomyocytes, compared to adult cardiomyocytes (Fig. 4a,b); similar responses are consistently reproduced following stimulation with CDCA and UDCA (Fig. 4c–f).

### Neither INT-777 nor UDCA reduce the contraction rate of NMVMs

**Figure 5:**
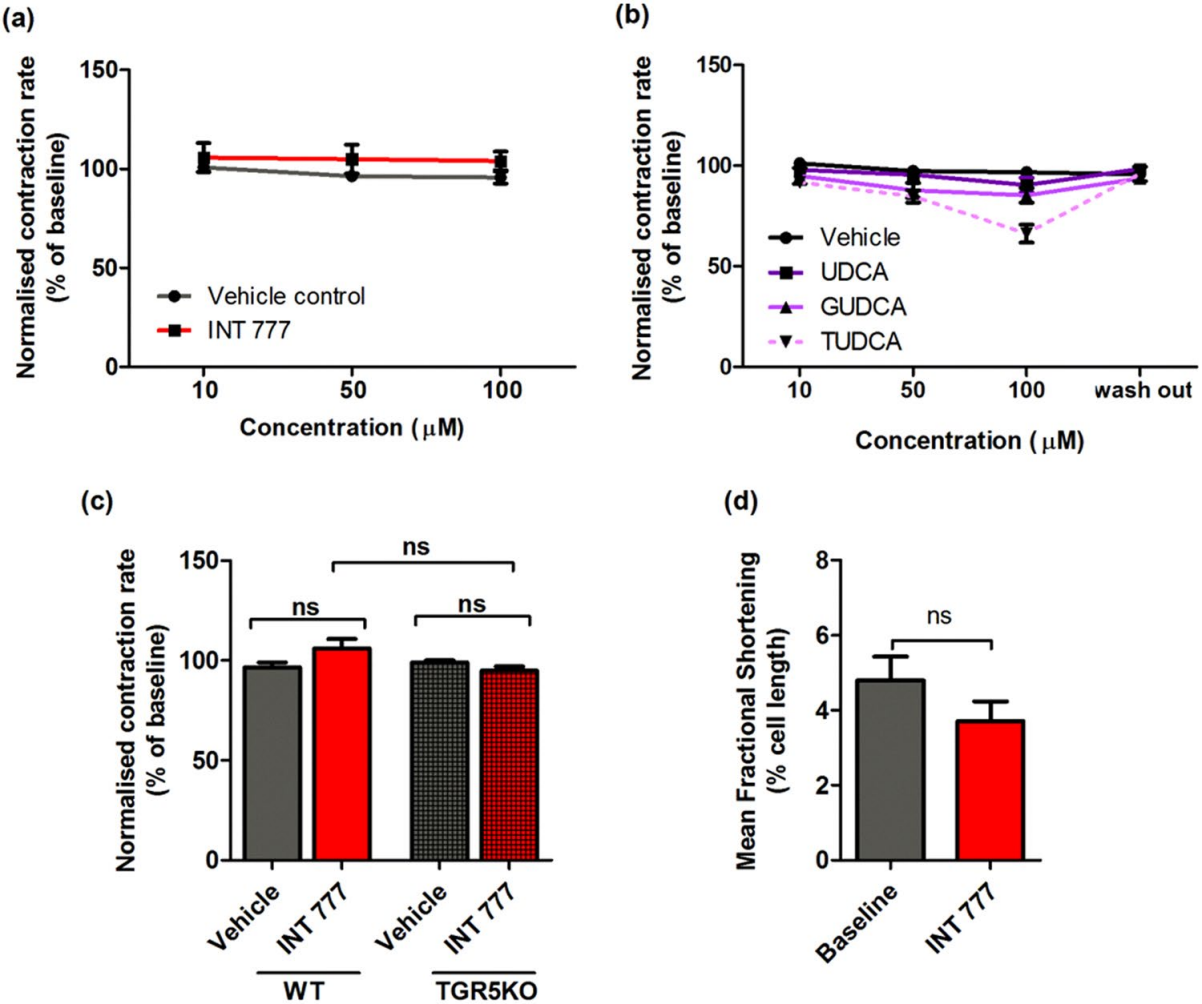
Ligands of TGR5 do not influence contraction of NMVMs. Contraction rate of NRVMs following treatment with INT77 (**a**) and UDCA and its conjugates (**b**) at 10–100 μM (n = 6, *p < 0.05, **p < 0.01); (**c**) Contraction of WT NMVMs in comparison to TGR5KO following treatment with INT 777 (100 μM) (n ≥ 6, ns, one-way ANOVA). (**d**) Effect of INT 777 (100 μM) on WT adult mouse cardiomyocytes contraction (n = 9, ns, unpaired t-test). All treatment were given acutely, following 10–15 mins perfusion. Data presented as mean ± SEM.

NMVMs in culture show spontaneous contraction. Incubation with INT-777, in the range of 10–100 µM, does not change the contraction rate of NMVMs in both wild type and TGR5KO cells (Fig. 5a,c). Similarly, UDCA and GUDCA do not alter the rate of NMVM contraction, and a non-significant trend for decreased contraction rate is seen with 100 µM TUDCA (Fig. 5b).

No change is observed in the fractional shortening (inotropic effect) of adult isolated mouse ventricular myocytes following incubation with INT-777 under electrical stimulation (Fig. 5d).

### Both un-conjugated and conjugated bile acids reduce the contraction rate of NMVMs

**Figure 6:**
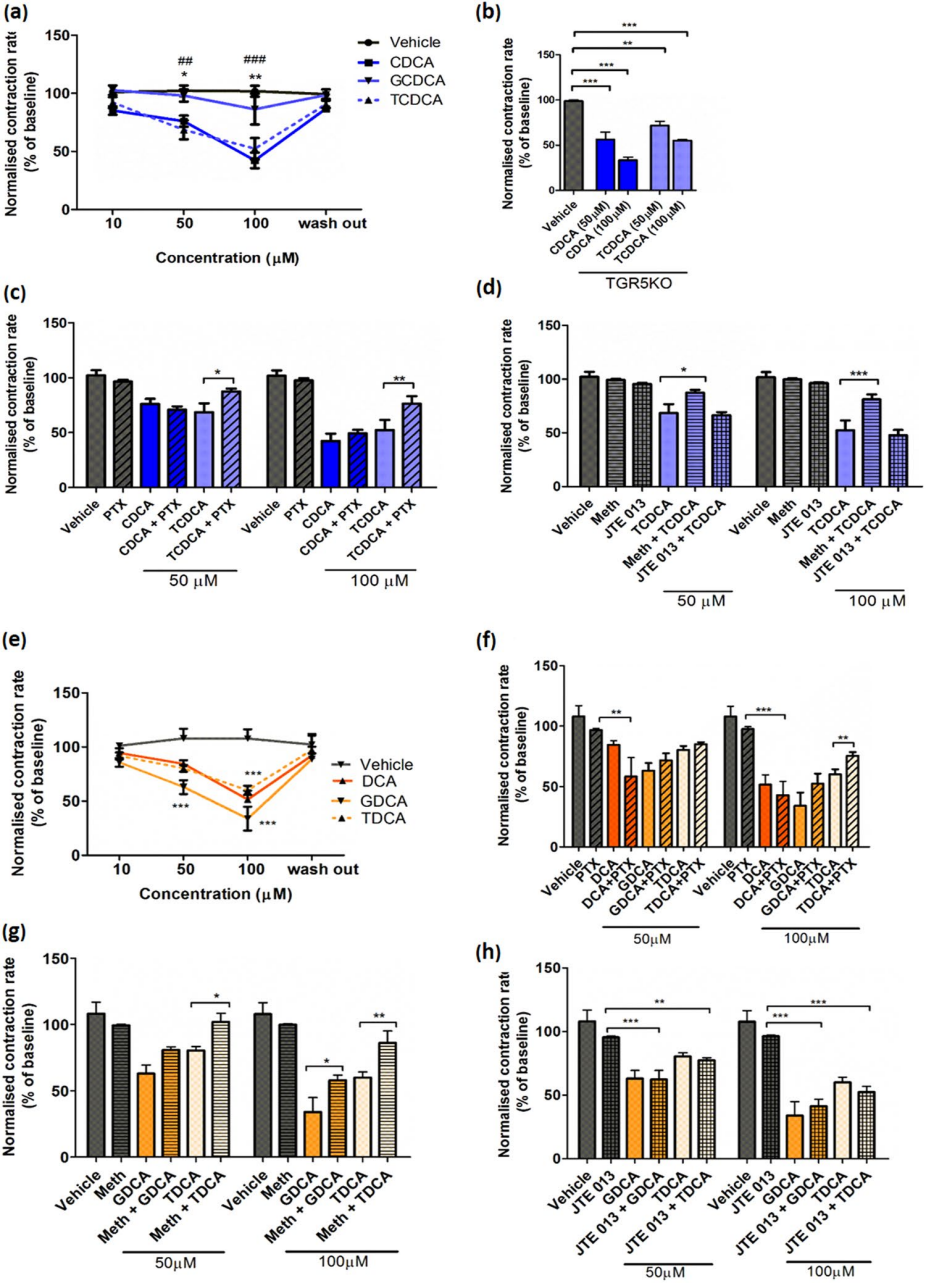
Dose-dependent reduction of contraction rate of NRVMs by conjugated bile acids depends on Gi signalling and muscarinic M2 receptor. (**a**) Contraction rate of WT NRVMs following treatment with CDCA and its conjugates at 10–100 μM (n = 6, *p < 0.05, **p < 0.01); (b) Contraction rate of WT and TGR5KO NRVMs following treatment with CDCA and TDCA at 50 and 100 μM (n = 5, **p < 0.01, ***p < 0.001); (**c**) Contraction rate of NRVMs with and without pretreatment with 1 µg/ml PTX followed by treatment with CDCA and TCDCA at 50 and 100 μM (n ≥ 5, *p < 0.05, **p < 0.01); (**d**) Contraction rate of NMVMs with and without pretreatment with either 1 µM methoctramine (M2 antagonist) or 10 µM JTE013 (S1P2 antagonist) followed by treatment with CDCA and TCDCA at 50 and 100 μM (n = 5, *p < 0.05, **p < 0.01); (**e**) Contraction rate of NMVMs following treatment with DCA and its conjugates at 10–100 μM (n = 6, *p < 0.05, ** < 0.01, two-way ANOVA with Benferroni post-hoc analysis). (f) Contraction rate of NMVMs with and without pretreatment with 1 µg/ml PTX followed by treatment with DCA and its conjugates at 50 and 100 μM (n ≥ 5, *p < 0.05, **p < 0.01, two-way ANOVA with Benferroni post-hoc analysis); (**g**) Contraction rate of NMVMs with and without pretreatment with 1 µM methoctramine (M2 antagonist) followed by treatment treatment with GDCA and TDCA at 50 and 100 μM (n ≥ 5, *p < 0.05, **p < 0.01, two-way ANOVA with Benferroni post-hoc analysis. (**h**) Contraction rate of NMVMs with and without pretreatment with 10 µM JTE013 (S1P2 antagonist) followed by treatment with CDCA and TCDCA at 50 and 100 μM (n ≥ 5, *p < 0.05, **p < 0.01, two-way ANOVA with Benferroni post-hoc analysis.

Unconjugated CDCA and DCA and conjugated TDCA, GDCA and TCDCA induce significant slowing in contraction at 50 and 100 μM (Fig. 6a,e), while GCDCA is less effective. Dose-dependent contraction slowing by CDCA and TCDCA is still present in TGR5 KO mice (Fig. 6b), further indicating that this receptor is not involved in the negative effects of bile acids, both conjugated and un-conjugated, on the NMVM contraction rate.

### Conjugated bile acids are partial agonists for the Gi pathway and muscarinic M2 receptor

At both 50 and 100 μM of all active conjugated bile acids tested (TCDCA, Fig. 6c, TDCA, and GDCA, Fig. 6f), pre-treatment with 1 µg/ml PTX, a Gi inhibitor significantly reduces the negative chronotropic effects. Thus, reduction in contraction triggered by conjugated bile acids is mediated through the Gi pathway, at least partially. However, this alleviation of the negative effect of bile acids is not present when CDCA (Fig. 6c) or DCA (Fig. 6f) were tested. If anything, slight exacerbation of the negative chronotropic effect of DCA (but not CDCA) is seen after PTX pre-treatment (Fig. 6f). The negative chronotropic effects of conjugated bile acids at 50 and 100 μM can also be partially eliminated following pre-incubation with the muscarinic M2 receptor antagonist, methoctramine (Fig. 6d,g). Hence, conjugated bile acids seem to act by binding to the M2 muscarinic receptor. In contrast, there is no such contractility change at both concentrations of bile acids (TCDA, GDCA and TCDCA) when NMVMs are pre-incubated with JTE 013, a S1P2 blocker (Fig. 6d,h). This indicates that conjugated DCA, TDCA and GDCA, do not exert their negative chronotropic effect through S1P2 receptor signalling.

### Prolonged incubation with conjugated bile acids and UDCA do not cause cytotoxicity in NMVMs

**Figure 7:**
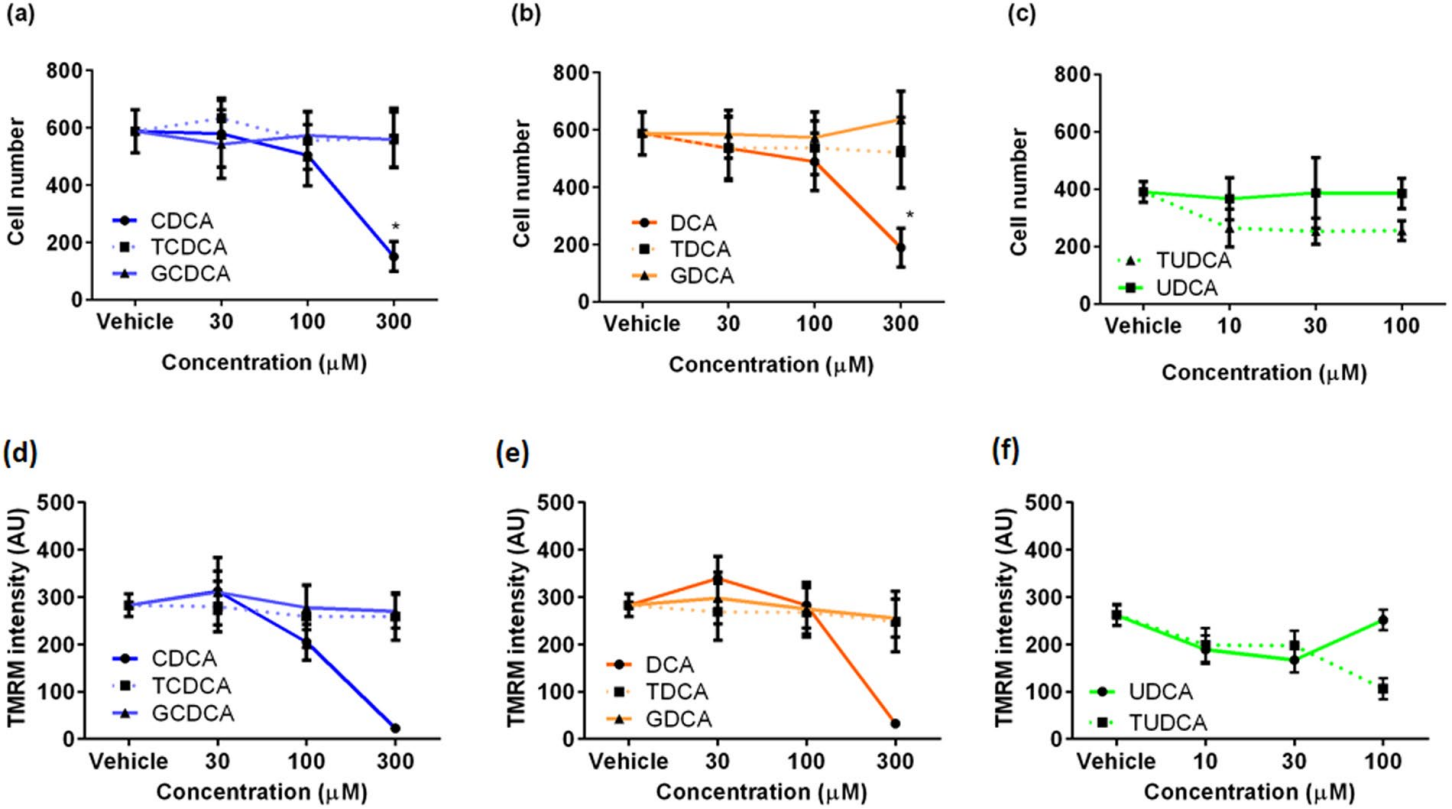
DCA and CDCA, but not UDCA and any conjugated bile acids, are toxic at high dose. Cell number following 24 hours treatment with (**a**) CDCA and conjugates; (**b**) DCA and conjugates; (**c**) UDCA and TUDCA at 30–300 μM (n = 5, *p < 0.05). Average intensity of TMRM following 24 hours treatment with (**d**) CDCA and conjugates; (**e**) DCA and conjugates; (**f**) UDCA and TUDCA at 30–300 μM (n = 5, *p < 0.05).

We established using high throughput toxicity assays that UDCA and all the conjugated bile acids do not damage mitochondria and do not exhibit toxicity towards cells. UDCA, TUDCA and the tauro- and glyco-conjugates of CDCA and DCA at all concentrations have no significant effect on cell count (Fig. 7a–c). Tauro- and glyco-conjugated CDCA, DCA and UDCA, as well as unconjugated UDCA, do not induce any mitochondrial damage at all concentrations tested (Fig. 7d–f).

### Prolonged incubation with high concentrations (300 µM) of unconjugated CDCA and DCA is toxic to NMVMs

In contrast to conjugated bile acids and UDCA, CDCA and DCA at 300 μM reduce NMVM numbers in comparison to control (Fig. 7a,b). Consistent with the cell count analysis, both un-conjugated CDCA and DCA at 300 μM also induce a dramatic decline in mitochondrial membrane potential (Fig. 7d,e).

## Discussion

Here we studied in detail the influence of a panel of primary and secondary bile acids and their conjugates, on the signalling and physiological outcomes in neonatal mouse ventricular myocytes (NMVMs). We found that TGR5 is expressed in both neonatal and adult cardiac myocytes and unconjugated BA are agonists for TGR5. In contrast, conjugated bile acids are not such potent agonists of TGR5 in NMVMs. This is an important observation, but unlikely to be of clinical relevance in the context of ICP because the principal BA in the fetal circulation are TCA, GCA, TCDCA and GCDCA^11^, there are very few unconjugated BA in the fetal circulation (although there is a slight increase in unconjugated UDCA in UDCA-treated pregnancies).

CDCA is a selective agonist for the nuclear receptor FXR, the most potent among endogenous bile acids^36,37^. We found this bile acid is also an agonist for TGR5 in NMVM, as judged by cAMP production. As TGR5 is the only known receptor of bile acids that is coupled with Gαs protein, we consider observing cAMP production to be a marker of TGR5 activation. Furthermore, the cAMP production following incubation with CDCA and UDCA is abolished in TGR5 knock-out mice (Fig. 3). Activation of TGR5 by CDCA is not surprising as bile acids are recognised as pluripotent molecules with broad reaction spectrum in a given tissue^38,39^.

Both conjugated and unconjugated bile acids have previously been described as potent agonists for TGR5^23,40^. These data were obtained with HeLa cells transfected with TGR5. In the constitutively expressing NMVMs we found only unconjugated bile acids can produce a substantial cAMP response. These differences may be related to the nature of downstream effectors present in HeLa cells compared to cardiac myocytes. This important finding supports the use of appropriate tissue-specific models to investigate the pharmacology of bioactive molecules with broad spectrum of effects.

Owing to the lack of effect of TGR5 activation upon contraction, the potential role of TGR5 in the neonatal heart remains to be ascertained. It may play a role in immune responses, metabolic alterations or stress tolerance, but no evidence is available at present.

The amount of cAMP produced by NMVMs in response to some bile acids, namely DCA UDCA and CDCA, equals that produced following stimulation with adrenaline or its synthetic analogue, isoprenaline (Fig. 1a,b). However, this doesn’t lead to increased contraction, both in terms of force or rate, in contrast with well-known isoprenaline effects^35^. This signifies that in cardiomyocytes the downstream signals from the beta-adrenoceptor and TGR5 are different. In addition, the signals may be in different subcellular compartments. Indeed, using localised FRET sensors we could show that TGR5 activates an adenylate cyclase isoform (RI) different from the RII isoform activated following adrenoceptor stimulation (Fig. 3).

On the other hand, conjugated bile acids at a relatively low concentration can reduce the NMVM contraction rate, at least partially, through the M2 receptor. Unconjugated CDCA and DCA also slow contraction, but independent of the M2 receptor and at a high concentration these compounds cause cytotoxicity and alter mitochondrial membrane potential.

Using inhibitor analysis, we did not observe any involvement of the S1P2 receptor. Pharmacological inhibition of the S1P2 receptor has been previously shown to significantly reduce the effect of conjugated bile acids in primary rat hepatocytes^28^. However, our investigation of the role of sphyngosin receptors was somewhat limited as we did not study S1P1, which is predominantly expressed in the heart^26^.

Conjugated bile acids were not effective at inducing cAMP production in our experiments. However, both unconjugated DCA and CDCA and their conjugates have a negative effect on NRVM contraction (Fig. 6). Previous research showed that GCDCA induces mitochondrial membrane permeability transition (MMPT) in a dose dependent manner in isolated rat hepatocytes^30,32^. However, in our experiments we did not see an adverse effect of any conjugated bile acids, including GCDCA, upon MMPT which probably reflects organ-specific variation or methodological differences.

An important clinical drug UDCA was found to be a potent agonist for TGR5 in NMVMs, whereas TUDCA and GUDCA are weak agonists (Fig. 2). This result is intriguing as UDCA has not been shown to activate TGR5 in previous studies^23^. We believe this novel finding is likely the consequence of bile acids having a different function in cardiomyocytes. At the same time neither UDCA nor GUDCA decrease contraction rate of NMVMs, and TUDCA has only small effect at high concentration (Fig. 4). Furthermore, UDCA and its conjugates do not show any toxicity even at high concentration. This is reassuring as UDCA is used to treat ICP and has been shown to cross the placenta^11^. Although unconjugated UDCA is present as a higher proportion of all bile acids in umbilical cord blood, as compared to maternal blood, unconjugated bile acids still constitute a small proportion of the bile acids identified^11^. We conclude therefore that Tgr5 is unlikely to play a role in fetal arrhythmia in the context of ICP.

**Figure 8:**
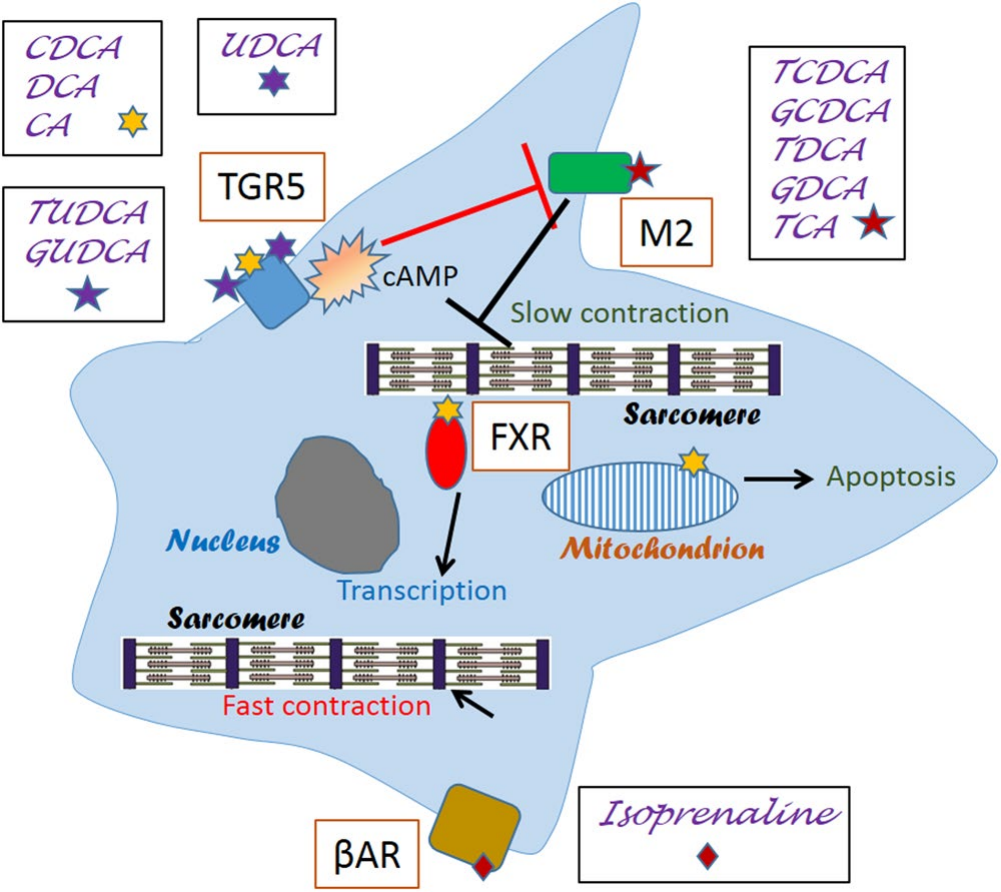
Schematic representation of signalling by various bile acids in NMVMs.

In summary, it appears that pleiotropic effects seen with many bile acids may be explained by the involvement of multiple mechanisms (Fig. 8). Our results indicate that the response of TGR5 to specific bile acids is different in NMVM than in the liver, intestinal L-cells or in other cell lines. Firstly, in NMVM UDCA activates TGR5 and not FXR or M2 receptors. Conjugated TUDCA and GUDCA are weak agonists for TGR5 and GUDCA does not activate M2, whereas TUDCA may partially activate it. This may result in a protective effect of UDCA against the action of TCA, which is an agonist for M2^16,41^. Conjugated bile acids TCDCA, GDCA, TDCA, GDCA and TCA bind to M2 receptors (and maybe other GPCRs), and this results in slowing of the rate of contraction. In NMVM unconjugated bile acids CDCA, DCA and CA are strong agonists for both TGR5 and FXR. However, any potential beneficial effect of CDCA and DCA activating TGR5 is masked by other mechanisms, for instance these bile acids also affect mitochondria and lead to toxicity when present at high concentration. As a result, both conjugated and unconjugated bile acids adversely influence contraction by slowing it (Figs 5 and 6) and, as was shown by us previously in the case of TCA, by inducing arrhythmias^5,7^.

In conclusion, we have delineated different mechanisms by which elevated serum bile acids in ICP may influence susceptibility to fetal arrhythmia. While raised unconjugated bile acid concentrations cause TGR5-mediated cAMP release in cardiomyocytes, this is not associated with an alteration in the rate of contraction. In contrast, conjugated bile acids do not impact TGR5, but they function as partial agonists of the M2 muscarinic receptor, with associated reduction in rate of contraction.

## Electronic supplementary material

supplementary figures
