# Supplementary material for: Bile acids and their respective conjugates elicit different responses in neonatal cardiomyocytes: role of Gi protein, muscarinic receptors and TGR5
Source: Sci Rep. 2018 May 8;8:7110. doi: 10.1038/s41598-018-25569-4 (PMC5940781; doi:10.1038/s41598-018-25569-4)
Supplement: Supplementary file 1 — supplementary figures [file 41598_2018_25569_MOESM1_ESM.pdf]

Supplementary information

Bile acids and their respective conjugates elicit different contraction and cAMP responses in neonatal cardiomyocytes: role of Gi protein, muscarinic receptors and TGR5.

Effendi Ibrahim, Ivan Diakonov, Dulasi Arunthavarajah, Teresa Swift, Mary Goodwin, Saraid McIlvride, Vanya Nikolova, Catherine Williamson, Julia Gorelik

(j.gorelik@imperial.ac.uk)

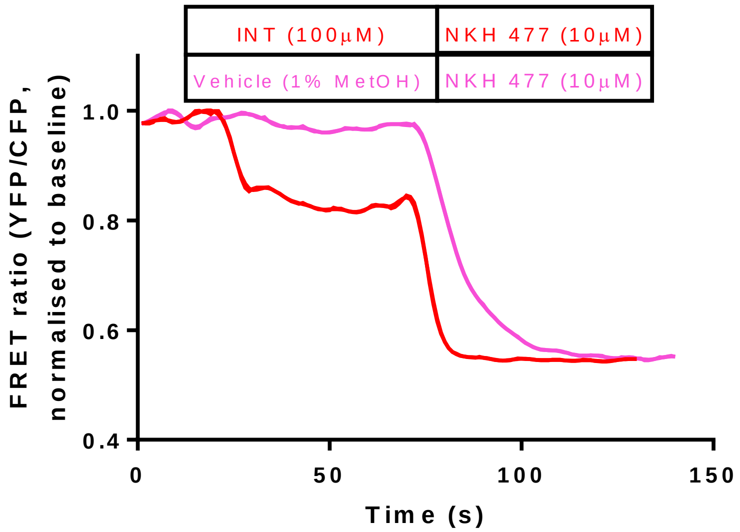

Figure S1. Vehicle control for FRET experiments with NRVMs. As most bile acids and INT77 were dissolved in methanol we checked FRET response for 1% methanol alone and did not find the response. (n=5)

(a)

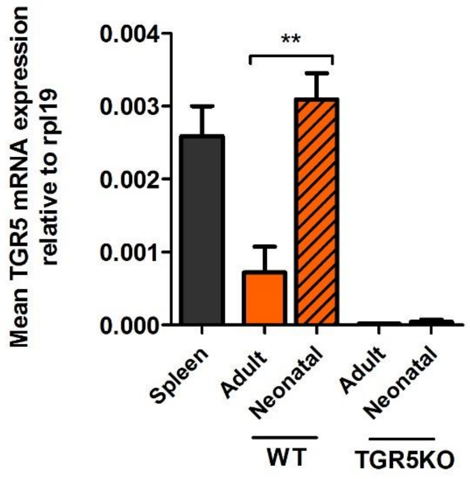

(b)

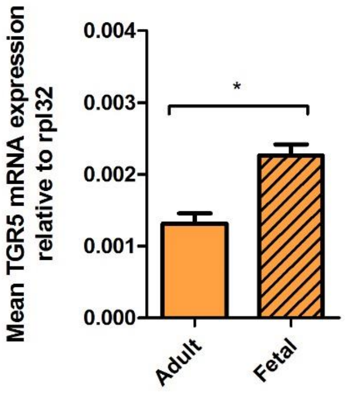

Figure S2. TGR5 mRNA expression in (a) heart tissue from adult and neonatal mice of WT and TGR5KO phenotype (n≥5, **p<0.01, one-ANOVA with Tukeys's post hoc analysis comparing all groups); (b) in human adult and fetal heart tissue (n ≥3, *p<0.05, Mann Whitney test. Data presented as mean ± SEM.

mRNA was isolated with the peqGOLD Total RNA Kit (PeqLab), adhering to the peqGOLD Total RNA Isolation Protocol. DNA was digested by DNase digestion (Qiagen). RNA concentration and purity was measured using the NanoDrop Spectrophotometer 8000 (ThermoFisher).

A reverse transcription, real time PCR was performed with standard protocol according to the manufacturer's inctructions (Qiagen, Eppendorf).
